# A novel graphene barrier against moisture by multiple stacking large-grain graphene

**DOI:** 10.1038/s41598-019-40534-5

**Published:** 2019-03-07

**Authors:** Ploybussara Gomasang, Kenji Kawahara, Kenta Yasuraoka, Mina Maruyama, Hiroki Ago, Susumu Okada, Kazuyoshi Ueno

**Affiliations:** 1 Graduate School of Engineering and Science, Shibaura Institute of Technology, Koto, Tokyo, 135-8548 Japan; 2 Global Innovation Center, Kyushu University, Kasuga, Fukuoka, 816-8580 Japan; 3 Graduate School of Pure and Applied Sciences, University of Tsukuba, Tsukuba, Ibaraki, 305-8577 Japan; 4 SIT Research Center for Green Innovation, Koto, Tokyo, 135-8548 Japan

## Abstract

The moisture barrier properties of stacked graphene layers on Cu surfaces were investigated with the goal of improving the moisture barrier efficiency of single-layer graphene (SLG) for Cu metallization. SLG with large grain size were stacked on Cu surfaces coated with CVD-SLG to cover the grain-boundaries and defective areas of the underneath SLG film, which was confirmed to be oxidized by Raman spectroscopy measurements. To evaluate the humidity resistance of the graphene-coated Cu surfaces, temperature humidity storage (THS) testing was conducted under accelerated oxidation conditions (85 °C and 85% relative humidity) for 100 h. The color changes of the Cu surfaces during THS testing were observed by optical microscopy, while the oxidized Cu into Cu_2_O and CuO was detected by X-ray photoelectron spectroscopy (XPS). The experimental results were accord with the results of first-principle simulation for the energetic barrier against water diffusion through the stacked graphene layers with different overlap. The results demonstrate the efficiency of SLG stacking approach against moisture for Cu metallization.

## Introduction

Copper (Cu) interconnects, which are used in large-scale integrated circuits (LSIs), require a barrier layer to prevent Cu diffusion, which leads to degradation in the Si carrier lifetime. As the interconnect size shrinks according to scaling, thinner barrier layer is required such as less than 1-nm thickness for beyond the 3-nm node to avoid increased resistance resulting from the reduced cross-sectional area of Cu^1^. Graphene, a two-dimensional carbon (C) layer, is a candidate for ultra-thin barrier layers because of its atomically thin crystal structure^2^. In addition to a potential diffusion barrier^3–6^, graphene has been reported as a candidate capping layer to enhance the electromigration (EM) reliability^7^, electrical conductivity, and thermal conductivity of Cu interconnects^8,9^.

Currently, LSIs are used primarily for data processing and have typical lifetimes of 10 years. LSIs are also expected to find applications in data storage as non-volatile and flash memories. These LSI memories could store data for more than 100 years since the digital data stored in LSIs are relatively easy to read compared to magnetic and optical storage media, which require specific tools to read the data. However, before LSIs can be applied in long-term storage, the reliability of Cu interconnects must be improved. To increase the lifetime of Cu interconnects, it is critical to develop ultra-thin protective barriers that prevent both the diffusion of both Cu and moisture. Graphene has been reported as a corrosion and diffusion barrier for metals such as Cu^10–12^, Ni^11,12^, Ag^13^, and Pt^14^. Graphene grown by CVD on Cu foil has been preliminarily demonstrated to investigate the moisture barrier properties of graphene^15–19^. We reported that CVD-grown single-layer graphene (SLG) can serve as an efficient impermeable film to prevent the diffusion of moisture. However, some areas of the Cu surface were oxidized^20^. The oxidized areas were speculated as the grain-boundaries or defective areas in the previous work.

In this study, two analytical experiments were performed. In the first experiment, we have confirmed that the oxidized areas in the SLG-coated Cu were corresponding to the grain-boundaries or defective structures of graphene by Raman spectroscopy measurements. For improvement in the resistance of graphene-coated Cu to prevent the oxidation from moisture, we report an artificial stacking SLG layers on CVD-SLG/Cu into double-layer graphene (DLG) to eliminate the Cu oxidation through the defects and grain boundaries of the underneath SLG, as in the second experiment. In addition, triple-layer graphene (TLG) was also formed by further stacking SLG on the DLG-coated Cu surface. To compare the oxidation resistances of DLG-coated Cu, TLG-coated Cu, SLG-coated Cu, and bare Cu surfaces, temperature and humidity storage (THS) tests were carried out under accelerated oxidation conditions. The sample surfaces were characterized using optical microscopy (OM) and X-ray photoelectron spectroscopy (XPS). The results indicate that the formation of DLG can improve the moisture barrier properties of the SLG-coated Cu surface by covering most of SLG grain boundaries. Moreover, TLG is a highly efficient barrier that uniformly prevents oxidation on the Cu film surface by covering the cross-points of grain boundaries in DLG. We discuss the mechanism of improvement by the first-principles simulation of overlapping graphene films result in an increased energetic barrier against the water diffusion through the different length of graphene overlap.

## Results

### Analysis of oxidized Cu areas on SLG-coated Cu surface

**Figure 1:**
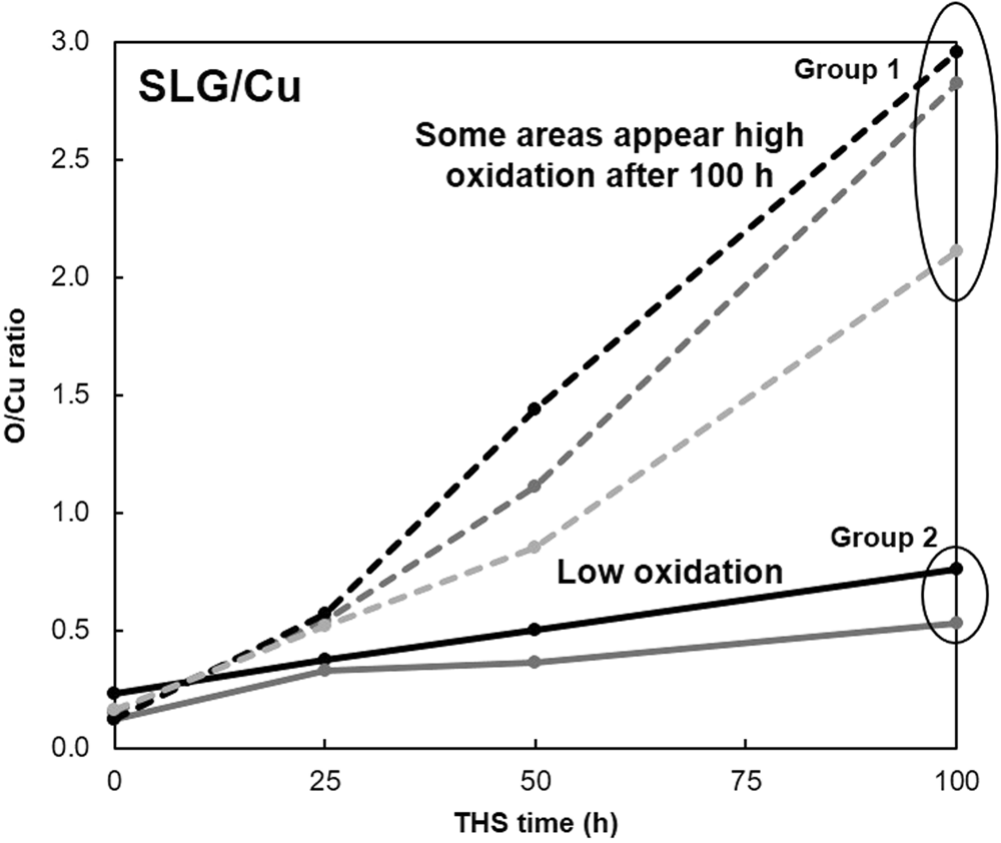
Relative O/Cu atomic concentration ratios of the SLG-coated Cu surfaces before and after different durations of THS testing.

**Figure 2:**
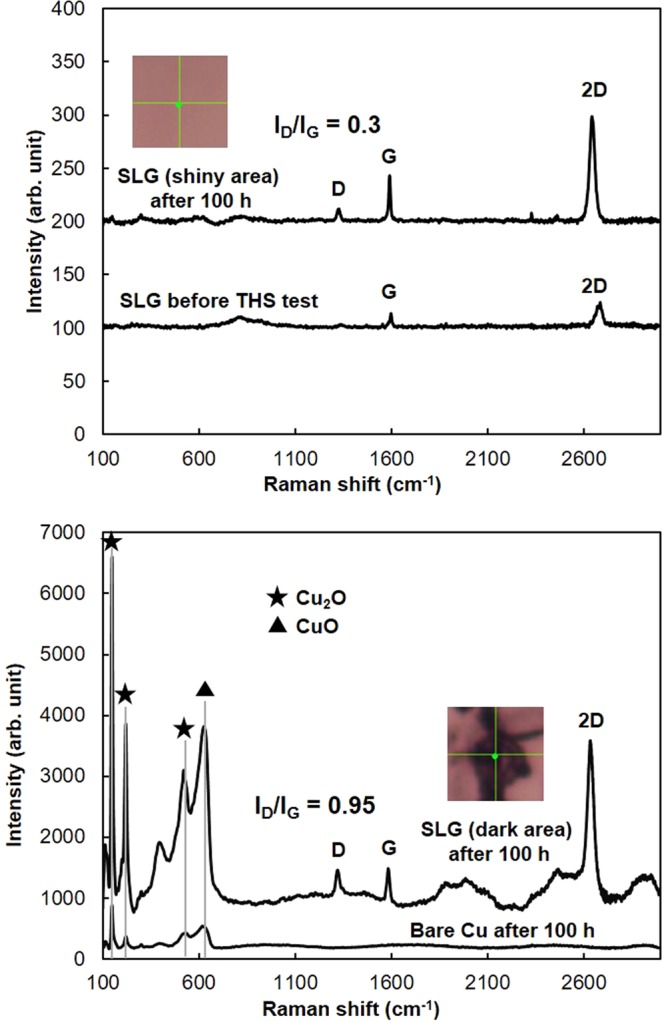
Raman spectra of graphene-coated Cu and bare Cu surfaces after 100 h of THS testing. Spectrum of SLG (shiny area) shows small D peak without Cu oxide peaks. Dark area of SLG and bare Cu spectra show different peak intensities of Cu_2_O and CuO depending on the density of Cu oxide present.

Regarding the previous report of moisture barrier properties of SLG-coated Cu surface^20^, we reproduce this experiment again, and then found that some areas of Cu surface were oxidized after 100 h of THS testing, as shown in the relative O/Cu atomic concentration ratios investigated by XPS (Fig. 1). In order to confirm the hypothesis that the oxidation occurred under grain boundaries or defective areas of SLG, dark and shiny areas on SLG-coated Cu surface were examined by Raman spectroscopy and also investigated the formation of Cu oxides comparing to those of bare Cu surface, as shown in Fig. 2. For the Raman spectra of SLG, the D peak, G peak, and 2D peak on SLG are located at 1320–1327, 1585–1597, and 2640–2680 cm^−1^, respectively^21,22^. The D peak corresponds to the defective structures of graphene. Comparing the D and G peaks intensity ratio (I_D_/I_G_) between the dark and shiny areas of SLG-coated Cu surface after 100 h of THS testing, the I_D_/I_G_ ratio of dark color area is relatively higher than that of shiny area. It indicates that there were more defects of the SLG such as grain-boundaries in the dark area than those of shiny area. In addition, the Raman spectra of the dark area exhibit peaks of Cu_2_O^23–25^ at 148, 219, and 525 cm^−1^, and the peak located at ∼630 cm^−1^ corresponds to CuO^26^. The intensities of both the Cu_2_O and CuO peaks are greater in the spectrum of the SLG-coated Cu surface compared to those of the bare Cu surface. The Raman spectrum measured on shiny areas of SLG-coated Cu surface after 100 h of THS testing do not show Cu oxide peaks. The results suggest that when graphene does not completely protect the Cu surface from moisture, the oxidation of the defective areas is enhanced by the graphene coating.

### Artificial stacking of SLG layers to cover the underneath grain boundaries

**Figure 3:**
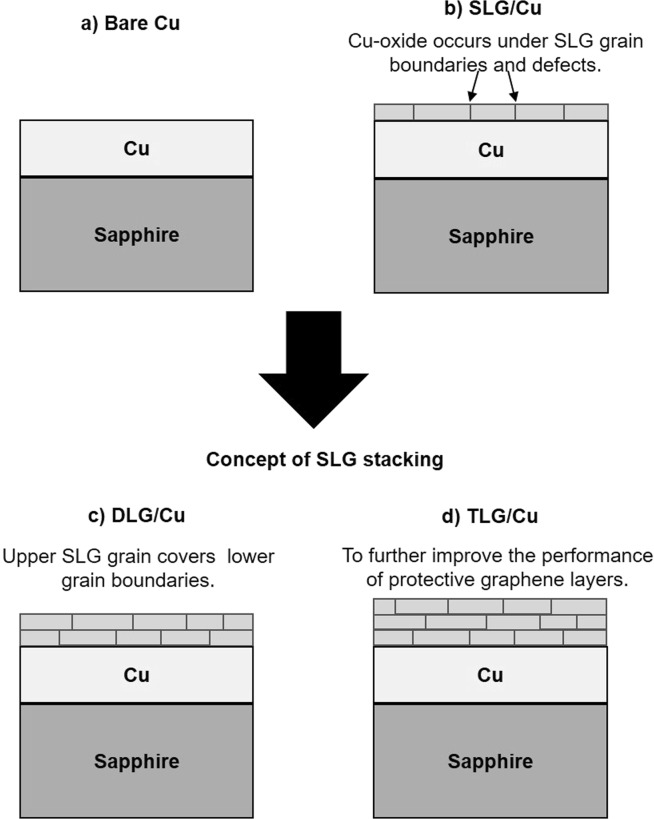
Structural models of the (**a**) bare Cu, (**b**) SLG-coated Cu, (**c**) DLG-coated Cu, and TLG-coated Cu samples. The models conceptually show how stacking SLG on Cu coated with CVD-deposited SLG and DLG layers covers the defects and grain boundaries of the underlying graphene layers.

In the second experiment, the oxidation resistance of SLG-coated Cu was improved by artificial stacking SLG layers. Figure 3 shows the concept of SLG stacking as DLG-coated Cu surface expected to eliminate Cu oxidation through the defects and grain boundaries in SLG-coated Cu surface, since the grain boundaries is expected to be covered by overlay SLG grains. Further SLG stacking on the DLG-coated Cu surface as TLG was also performed to improve the integrity of covering grain-boundaries and defects.

#### Change of surface color during THS testing observed by OM

**Figure 4:**
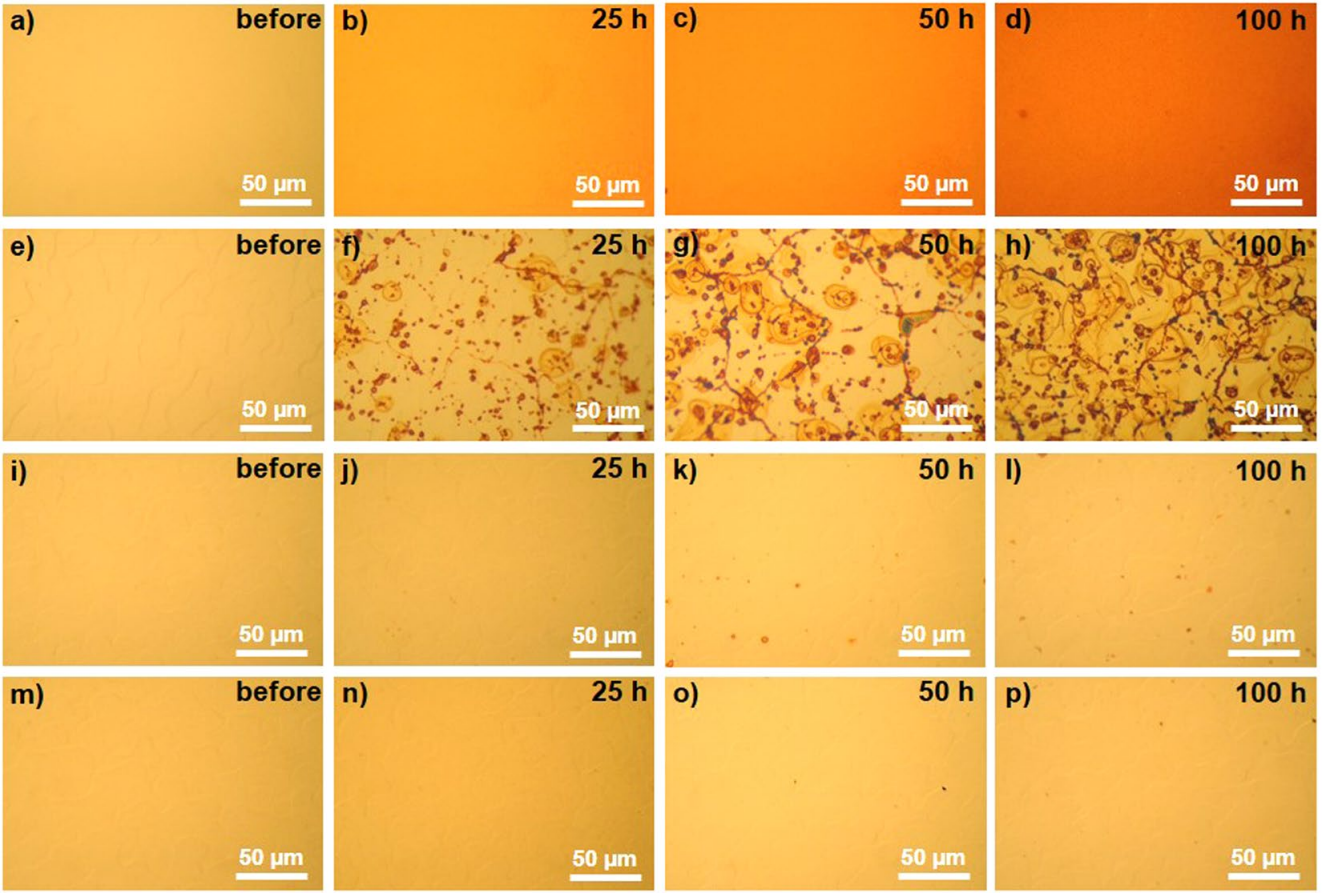
OM images of sample surfaces before and after different durations of THS testing. OM images of (**a‒d**) bare Cu, (**e‒h**) SLG-coated Cu, (**i‒l**) DLG-coated Cu, and (**m‒p**) TLG-coated Cu surfaces.

All samples were prepared, including SLG-coated Cu and bare Cu samples, and then tested in THS chamber. Primary observation in the color changes of the bare Cu and graphene-coated Cu surfaces were carried out by OM with comparing before and after variously THS testing times (Fig. 4). Compared to its initial state (Fig. 4a), the bare Cu surface rapidly changed to a uniformly dark color after 25 h of THS testing (Fig. 4b) and became continually darker as the long-term THS test continued (Fig. 4c,d). The changes in color indicate that the Cu surface was oxidized upon exposure to high temperature and humidity. Most areas of the SLG-coated Cu surface appeared shiny after THS testing (Fig. 4f), similar to the initial surface before testing (Fig. 4e). However, in some areas, dark lines and spots appeared (Fig. 4f‒h) and became larger and darker after long-term THS testing (Fig. 4b−d). The color change of SLG-coated Cu surface was qualitatively similar to the previous report^20^, but the dark color areas were relatively larger in this study probably due to the higher density of defects. In contrast, the DLG- and TLG-coated surfaces exhibited large shiny areas after THS testing. These results support our expected effects of stacking SLG layers cover relatively defective areas of underneath SLG. Although some small dark spots were observed on the DLG-coated Cu surface after long-term THS testing, the shiny areas on the DLG-coated Cu surface were clearly larger (Fig. 4j‒l) than those on the SLG-coated Cu surface (Fig. 4f‒h). The dark spots were nearly eliminated on the TLG-coated Cu surface, and a uniformly shiny surface without dark spots and lines was observed after 100 h of THS testing (Fig. 4n‒p).

#### Evolution of Cu oxides during THS testing measured by XPS

**Figure 5:**
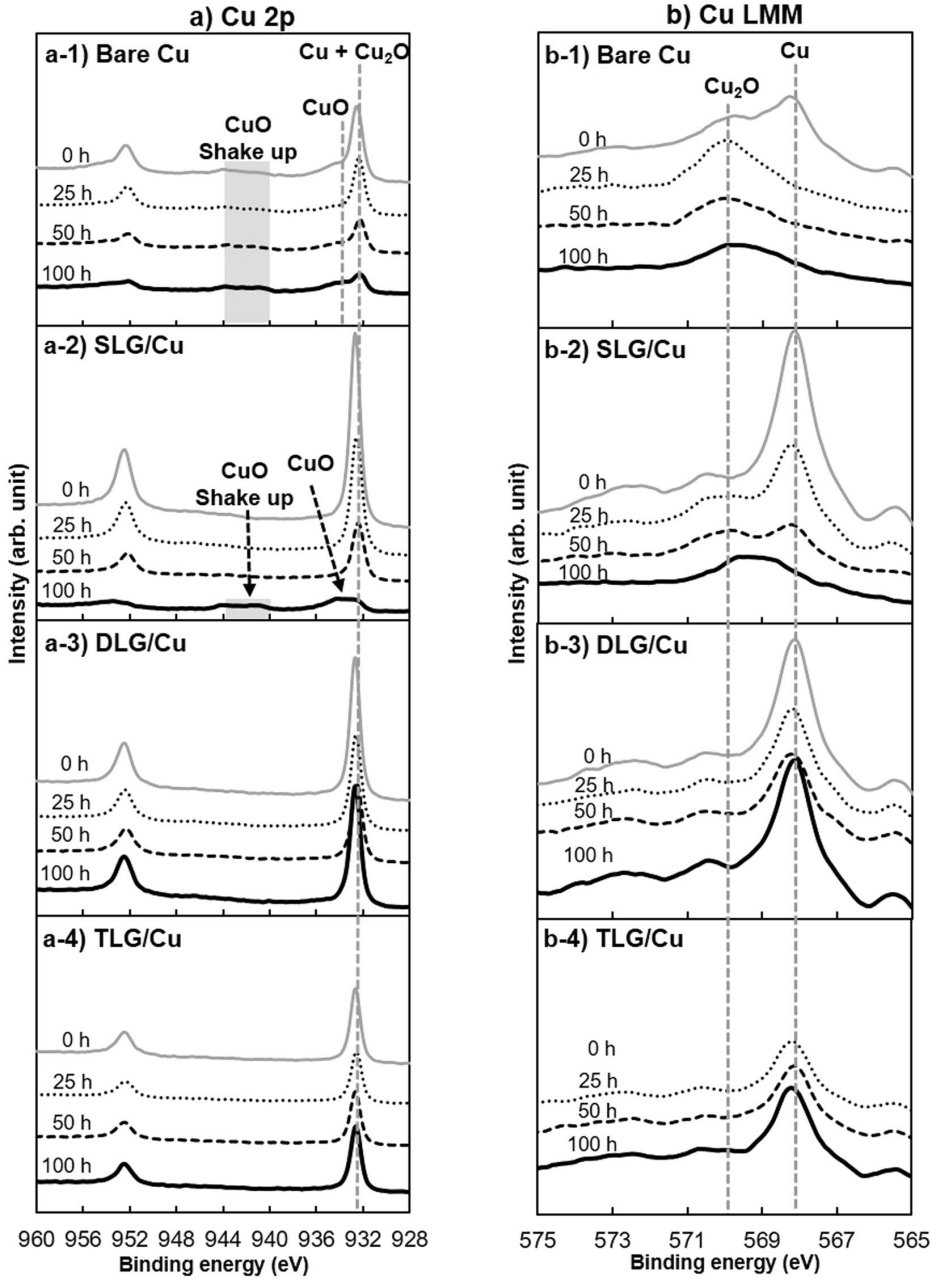
XPS spectra of bare and graphene-coated Cu surfaces before and after different durations of THS testing. Panels (a) and (b) respectively show the Cu 2p and Cu LMM XPS spectra to analyze the presence of metallic Cu, Cu_2_O, and CuO on each sample surface.

The relative changes in the contents of O and Cu during THS testing were evaluated by XPS. A typical spectrum from each duration was exhibited, as shown in Fig. 5. The XPS peak intensity is associated with the average amount of the corresponding elements within the analyzed XPS depth. The primary analysis confirmed the components of metallic Cu, Cu_2_O, and CuO in the Cu 2p spectrum, as shown in Fig. 5(a-1–a-4). The main peaks observed from 932.6‒932.4 eV correspond to metallic Cu and the low oxidation state of Cu_2_O^27–30^. The high oxidation state of CuO is reflected by the peaks at ∼933.6 eV^27–29^, and its satellite peak is observed from 944.6‒942.4 eV^27,28^. The peaks of metallic Cu and Cu_2_O can be distinguished in the Cu LMM Auger spectrum, as shown in Fig. 5(b-1‒b-4).

The Cu 2p spectrum of the bare Cu surface before THS testing [Fig. 5(a-1)] exhibits high-intensity peaks of metallic Cu and Cu_2_O along with small CuO and CuO satellite peaks. In the Cu LMM Auger spectrum of the bare Cu surface, the peaks at the binding energies of ∼567.9 and ∼570 eV correspond to metallic Cu^31^ and Cu_2_O^17^, respectively. The Cu LMM spectrum of bare Cu before THS testing [Fig. 5(b-1)] exhibits peaks of both metallic Cu and Cu_2_O at 0 h, indicating that a layer of Cu_2_O formed on the Cu surface upon exposure to air before the THS test. After THS testing, the peaks corresponding to metallic Cu and Cu_2_O in the Cu 2p spectrum [Fig. 5(a-1)] decreased continually, whereas the CuO peak increased, indicating that Cu continued to be oxidized during the THS test. No metallic Cu was observed after 100 h of testing, as shown in the Cu LMM Auger spectrum [Fig. 5(b-1)].

The Cu 2p spectra of the SLG-, DLG-, and TLG-coated Cu surfaces before THS testing [Fig. 5(a-2‒a-4)] exhibit high-intensity peaks of metallic Cu and Cu_2_O. In the Cu LMM Auger spectra [Fig. 5(b-2‒b-4)], the peak intensity of metallic Cu is much higher than that of Cu_2_O. These results demonstrate the ability of the graphene layers to protect the Cu surface. Significant changes were observed in the Cu 2p spectrum of the SLG-coated Cu surface after long-term THS testing [Fig. 5(a-2)]. The peak of CuO appeared suddenly after 100 h of testing, and its intensity was greater than that of the Cu_2_O peak. This change coincides with the appearance of dark lines and spots, as observed by OM (Fig. 4h). The Cu 2p spectra of the DLG- and TLG-coated Cu surfaces [Fig. 5(a-3,a-4)] show strong peaks corresponding to metallic Cu + Cu_2_O components, whereas the CuO peaks are almost nonexistent. The Cu LMM Auger spectra of the DLG- and TLG-coated Cu surfaces [Fig. 5(b-3,b-4)] clearly indicate the presence of a large amount of metallic Cu after 100 h of THS testing, in agreement with the large shiny areas observed on the Cu surfaces by OM [Fig. 4(i‒p)].

**Figure 6:**
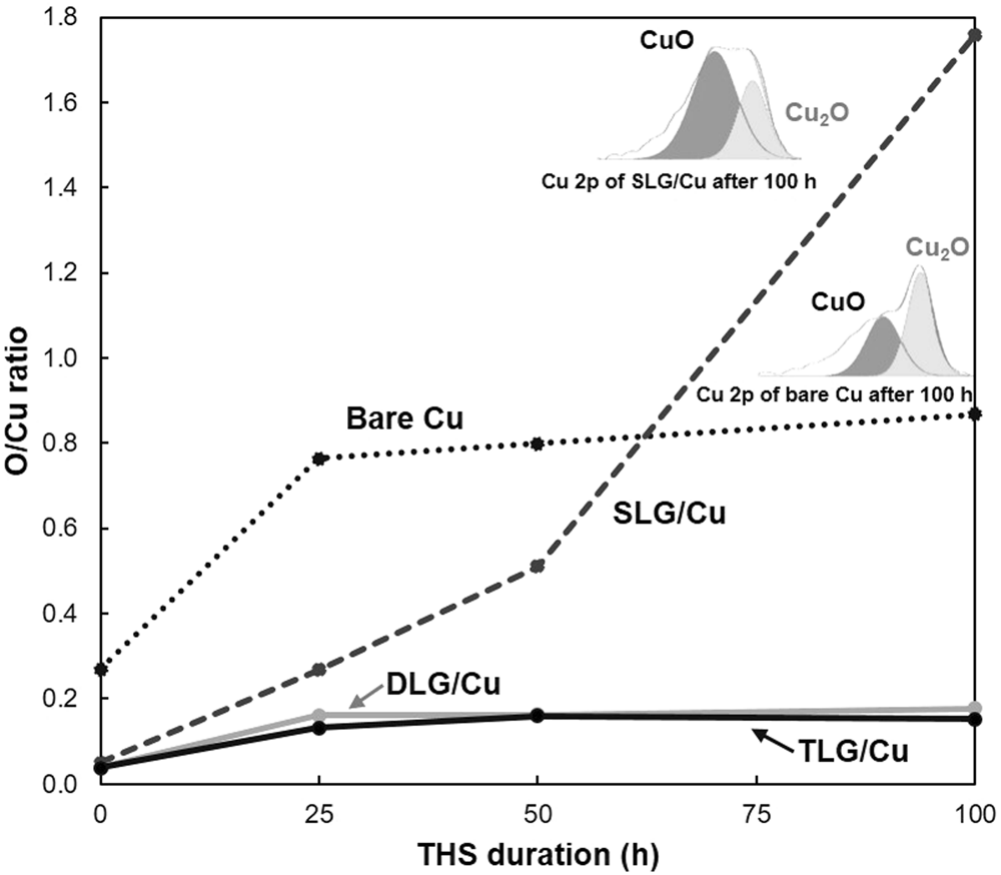
Relative O/Cu atomic concentration ratios of the bare Cu and graphene-coated Cu surfaces before and after different durations of THS testing.

Figure 6 shows the relative O/Cu atomic concentration ratios estimated from the Cu 2p and O 1 s spectra; adsorbed O^32,33^ and C–O bonds^32^ were not included when determining the concentration ratios (Fig. S1 in Supplementary Information). Before THS testing, the O/Cu ratio of the bare Cu surface was higher than those of all the graphene-coated Cu surfaces because the bare Cu surface was oxidized in air. The rate of increase in the O/Cu ratio on the bare Cu surface was maximized after 25 h of the THS testing and then decreased as the test continued. For the SLG-coated surface, the O/Cu ratio increased linearly with THS testing time and then increased extremely quickly after 100 h of testing due to the higher content of CuO than Cu_2_O comparing to those of bare Cu surface, as shown in inset Cu 2p curve fitting. These results agree with the dark lines and spots investigated on the sample surface by Raman spectroscopy (Fig. 2) and the change in shape of the Cu 2p spectra (Fig. 5). For the DLG- and TLG-coated Cu surfaces, the O/Cu ratios increased slightly after 25 h of THS testing and then remained stable.

## Discussion

**Figure 7:**
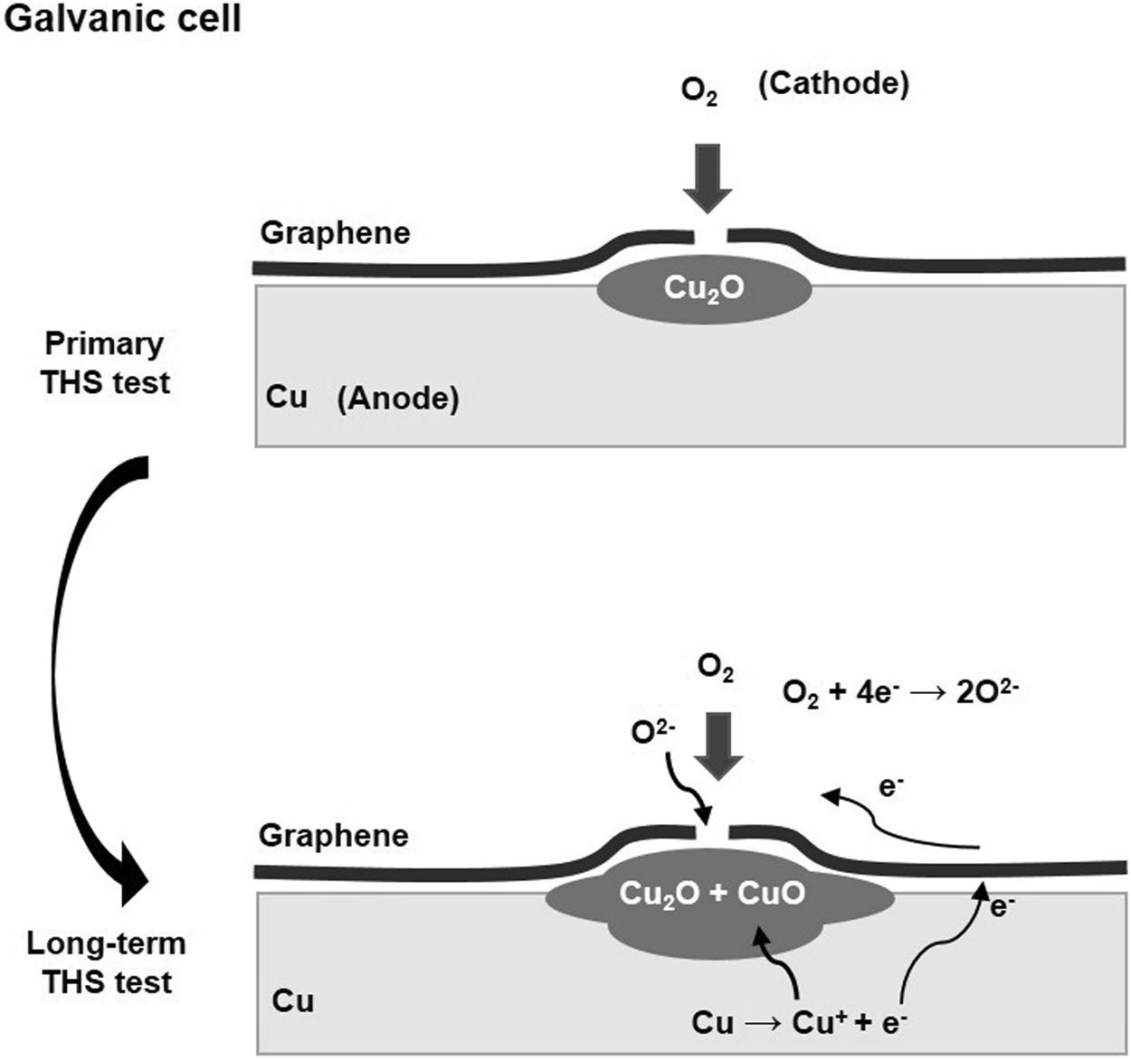
Schematic model showing the formation of a galvanic cell on a graphene-coated Cu surface.

Coating the Cu surface with a high-quality SLG film generated a moisture barrier that prevented the oxidation of the Cu film, as indicated the shiny areas observed by OM on the SLG-coated Cu surface (Fig. 4f‒h). However, some areas of SLG-coated Cu surface show dark color owning to the formation of Cu oxides under the grain boundaries and defective areas of SLG layers, as indicated by Raman spectroscopy (Fig. 2). A comparison of oxidation between the bare Cu and SLG-coated Cu surfaces by Raman spectroscopy (Fig. 2), OM images (Fig. 4b‒d,f‒h) and XPS results (Figs 5, 6) reveals that SLG could preserve the metallic Cu surface underneath graphene during 50 h of THS testing; however, the efficiency of the SLG coating was reduced after long-term THS testing (100 h). In addition, Raman spectroscopy revealed the enhanced Cu oxidation at the sites of defects and grain boundaries of SLG compared to on the bare Cu surface (Fig. 2). This enhanced oxidation can be explained by the formation of a galvanic cell^34^, as shown schematically in Fig. 7. When Cu_2_O begins to form on unprotected areas of the Cu surface, a galvanic cell is formed. This cell is in contact with conductive graphene, which transfers electrons to O, thereby promoting further Cu oxidation at SLG defects and grain boundaries. The results indicate that the oxidation rate of the bare Cu surface decreased after long-term THS testing because no galvanic cell was formed to accelerate Cu corrosion.

**Figure 8:**
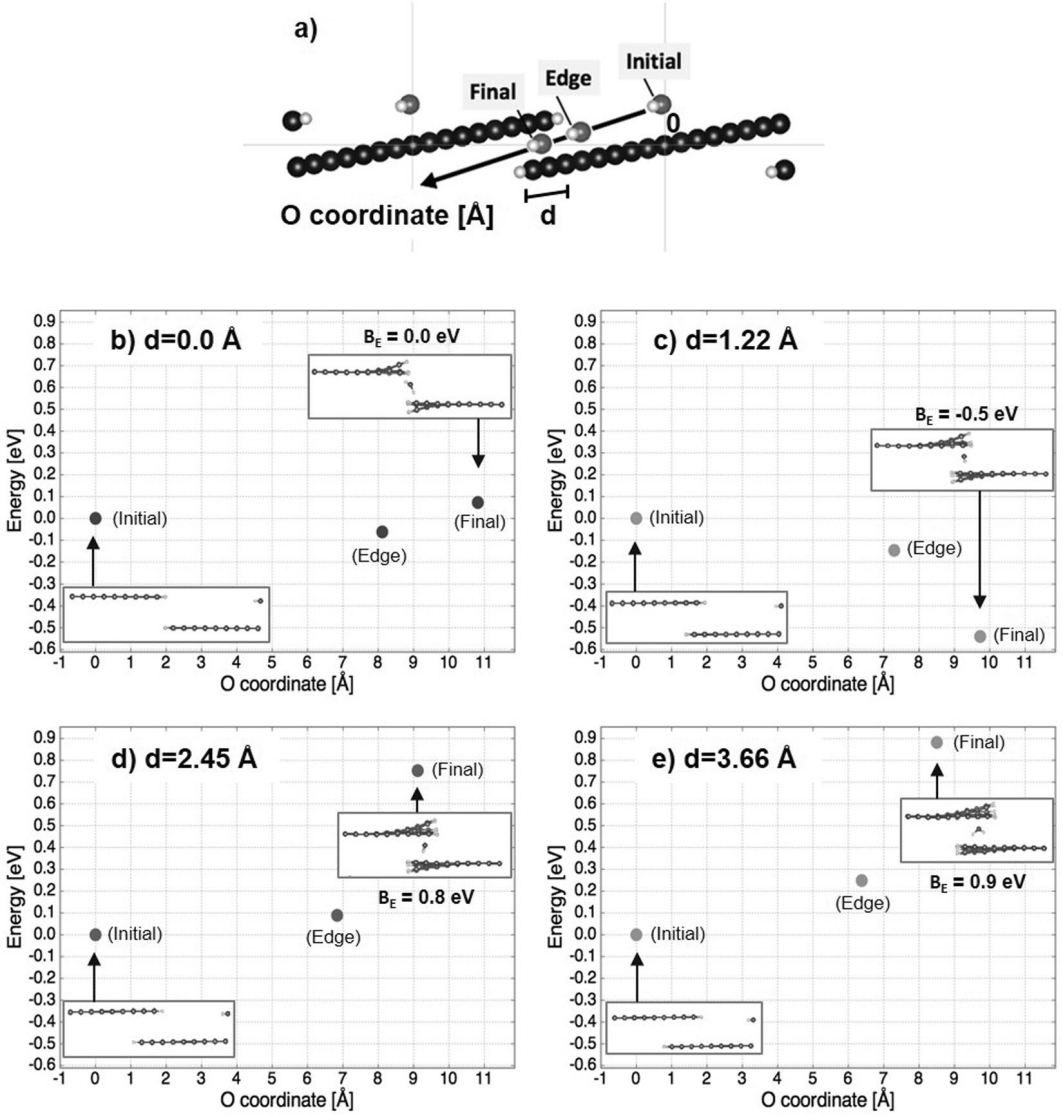
Optimized geometry and energy of water migration through overlapping areas of graphene nanoribbons. Panel (a) shows the migration coordinate of O. Panels (b‒e) show the water migration energies (*E*) for different lengths of graphene overlap (*D* = 0, 1.22, 2.45, and 3.66 Å, respectively).

To eliminate the oxidation at the sites of SLG grain boundaries and defects, we studied the effect of covering over the grain boundaries and defects by stacking SLG layer as DLG. As expected, DLG-coated Cu surface exhibited larger shiny areas after long-term THS testing (Fig. 4j‒l) because the water passing through the SLG defects and grain boundaries was obstructed by the additional layer of graphene. Little change was observed in the XPS spectrum of DLG-coated Cu during 100 h of THS testing [Fig. 5(a-3)], indicating good protective ability. To help explain how the upper graphene layer protects areas with grain boundaries or defects, Fig. 8 shows the simulated energy barrier to water migration through overlapped graphene layers. In the first-principle simulation, migrating water moved from the middle-right side to left side of both graphene layers, and water migrates along the migration coordinate via the edge site and ends among the overlapped graphene layers (Fig. 8a). The simulation results indicate that the energy barrier (*E*) to water migration was higher when the length of graphene overlap (*D*) was 1.22 to 3.66 Å (Fig. 8c‒e) compared to the scenario with no overlap (*D* = 0 Å; Fig. 8b). The results indicate that graphene overlap is essential to improve the barrier efficiency.

**Figure 9:**
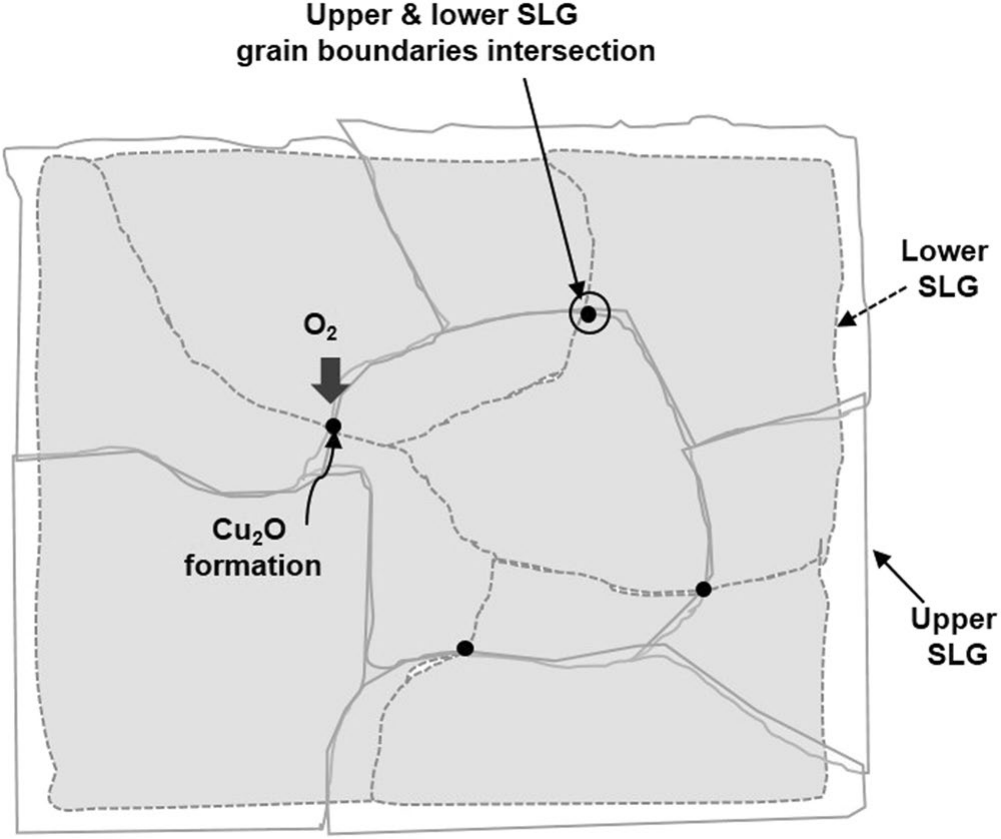
Schematic model showing the likely cause of Cu oxidation on the DLG-coated Cu surface.

However, small dark spots of Cu_2_O and CuO were still observed on some areas of the DLG-coated Cu surface, as indicated by OM (Fig. 4j‒l). These tiny dark spots, which were all similar in size, likely correspond to the intersection between the upper and lower SLG grain boundaries, as shown in the schematic model in Fig. 9. The O atoms can penetrate through these holes to oxidize Cu surfaces.

The moisture barrier properties were further improved by stacking SLG on the DLG-coated Cu surface to form TLG. The number of small dark spots on the TLG-coated surface after 100 h of THS testing (Fig. 4n‒p) was drastically reduced compared to on the DLG-coated Cu surface (Fig. 4j‒l), likely because the additional stacked SLG covered the defects of the lower graphene layers. The Cu 2p spectrum and O/Cu ratio of the TLG-coated surface did not change significantly during THS testing [Figs 5(a-4), 6], confirming the ability of TLG to prevent the oxidation of the Cu surface by moisture. Overall, the findings demonstrate that stacking graphene layers can effectively prevent the diffusion of moisture through the defects and grain boundaries of the lower graphene layers.

## Conclusion

Raman spectroscopy measurement on the oxidized areas of the SLG-coated Cu surface and XPS analysis clearly suggests that the obstruction of grain boundaries and defective areas of SLG should be concerned not only to prevent the oxidation on Cu but also to eliminate the galvanic cell formation, which accelerates high oxidation in long-term storage. To improve the moisture barrier properties of large-grain SLG on Cu(111)/sapphire substrates, stacking SLG on the SLG-coated Cu surface was performed to cover defects and grain boundaries of the underlying SLG surface. Long-term THS testing (100 h) was carried out under accelerated oxidation conditions (85 °C and 85% relative humidity) to evaluate the effectiveness of the graphene coatings at preventing oxidation. The change on sample surfaces were analyzed by OM and XPS. The results show that SLG stacking as DLG was an efficient barrier against O diffusion, although some small areas of Cu were still oxidized at the intersections of grain boundaries between the upper and lower SLG layers. Further stacking as TLG-coated Cu surface can be achieved to preserve the Cu film surface in long-term THS testing. The simulation results indicate that energy barrier against water migration was higher with increasing the graphene overlap. The results indicate that it is essential to stack for covering the underneath grain boundaries and defects, not just stacking the SLG layers. The findings demonstrate that stacking large-grain SLG is a promising strategy for improving the moisture barrier properties of graphene on Cu film surface.

## Methods

Large-area SLG was grown on Cu/sapphire substrates using a previously reported CVD method^35,36^. First, a 1000-nm-thick Cu(111) film was deposited on a c-plane sapphire substrate via high-temperature sputtering. To deoxidize the Cu surface before SLG deposition, the Cu/sapphire substrate was annealed in a flow of H_2_ and Ar gases at 1000 °C for 40 min. The temperature was then increased to 1075 °C over 20 min. SLG growth occurred on heteroepitaxial Cu (111)/sapphire substrates via ambient-pressure CVD at 1075 °C in CH_4_ precursor gas. The quality of the SLG film was confirmed by Raman spectroscopy after transferring the film to a SiO_2_/Si substrate (Fig. S2 in Supplementary Information). DLG- and TLG-coated Cu samples were prepared by stacking SLG on SLG/Cu samples formed by CVD using transfer method^37^. A bare Cu sample was also deposited on a sapphire substrate by sputtering and annealing in a quartz tube; to facilitate comparison, the procedure and conditions used to deposit the bare Cu sample were identical as those used for SLG growth by CVD but without the CH_4_ precursor gas.

THS testing was conducted for 100 h in a THS chamber under conditions designed to accelerate oxidation (85 °C and 85% relative humidity). The first experiment was carried out to examine the oxidized areas in the SLG-coated Cu surface after 100 h of THS testing measured by Raman spectroscopy measurement at an excitation wavelength of 633 nm and spot diameter of ~1 μm. The second experiment was performed for testing the efficiency of stacking graphene samples, comparing to SLG-coated Cu and bare Cu samples. Before THS testing and after 25, 50, and 100 h of test, all sample surfaces were observed by OM. Cu 2p, Cu LMM, and O 1 s XPS spectra were collected using an Al X-ray source (1486.6 eV) with a spot diameter of ~110 μm. The O/Cu atomic concentration ratio was evaluated from the O 1 s and Cu 2p spectra after background subtraction.

Unstitched grain boundaries of graphene were simulated for armchair graphene nanoribbons with hydrogenated edges. Water migrated through the overlapping graphene areas was simulated with various overlapped lengths (*D* = 0, 1.22, 2.45, and 3.66 Å). The O atom of water was fixed along the migration coordinate. Water migration began from the center of the graphene nanoribbon and ended among the overlapped graphene layers via the edge site. The energy barrier to water migration was estimated form the simulation results.

## Supplementary information

Supplementary Information
