# Supplementary material for: A novel graphene barrier against moisture by multiple stacking large-grain graphene
Source: Sci Rep. 2019 Mar 7;9:3777. doi: 10.1038/s41598-019-40534-5 (PMC6405749; doi:10.1038/s41598-019-40534-5)
Supplement: Supplementary file 1 — Supplementary Information [file 41598_2019_40534_MOESM1_ESM.pdf]

Supplementary Information

A novel graphene barrier against moisture by multiple stacking large-grain graphene

Ploybussara Gomasang [1], Kenji Kawahara [2], Kenta Yasuraoka [3], Mina Maruyama [3], Hiroki Ago [2], Susumu Okada[3], and Kazuyoshi Ueno [1, 4,*]

[1] Graduate School of Engineering and Science, Shibaura Institute of Technology, Koto, Tokyo 135-8548, Japan

[2] Global Innovation Center, Kyushu University, Kasuga, Fukuoka 816-8580, Japan

[3] Graduate School of Pure and Applied Sciences, University of Tsukuba, Tsukuba, Ibaraki, 305-8577, Japan

[4] SIT Research Center for Green Innovation, Koto, Tokyo 135-8548, Japan

*ueno@shibaura-it.ac.jp

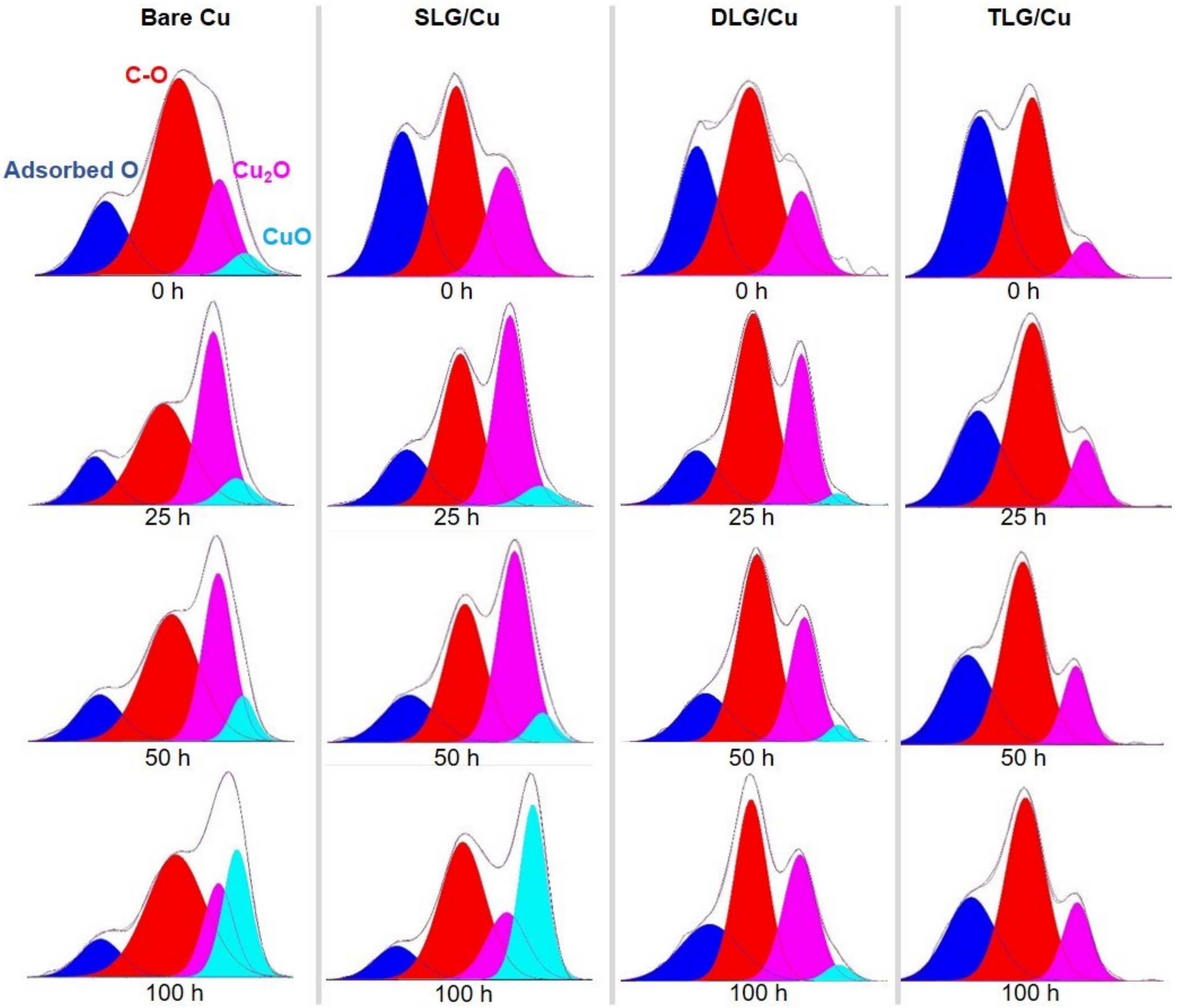

Supplementary Figure S1. O 1s curve fittings showing the amount of O atom in Cu_2O, CuO, C–O bonds, and adsorbed O on each sample surface. The evolution of O atoms in the Cu_2O and CuO components of the O 1s spectra corresponds to the analysis of the Cu 2p spectra. Adsorbed O and C–O components were omitted in the calculation of O/Cu ratios.

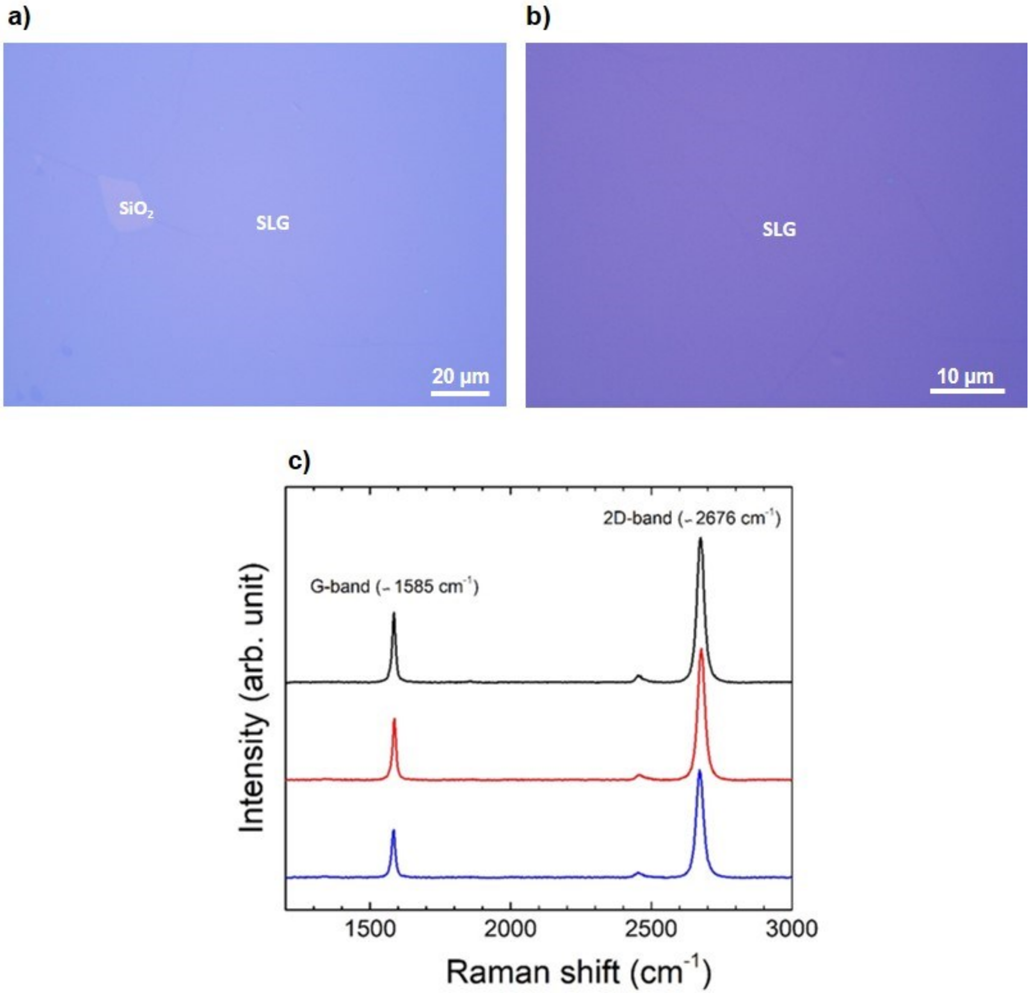

Supplementary Figure S2. Characterization of SLG. Panels (a and b) show Optical micrographs of SLG grown on Cu(111)/sapphire substrate after transferring the film to a SiO_2 (300 nm)/Si substrate. For panel (a), we chose the area with a hole showing bare SiO_2 to demonstrate that the growth of uniform SLG, almost free from multilayers. Panel (c) shows Raman spectra measured on the transferred SLG surface (excitation wavelength of 532 nm).
